# Mediterranean Journal of Rheumatology December 2017 Highlights

**DOI:** 10.31138/mjr.28.4.169

**Published:** 2017-12-22

**Authors:** Theodoros Dimitroulas

**Affiliations:** Aristotle University of Thessaloniki, Thessaloniki, Greece

On behalf of the Editorial Board, I welcome you to the December 2017 issue of the *Mediterranean Journal of Rheumatology*. In the next pages, you will find interesting papers covering a wide spectrum of topics including education in Rheumatology, recent data for targeted synthetic disease*-*modifying antirheumatic drug*s* in psoriatic arthritis*,* metabolic bone disease, physical therapy, pathogenetic pathways of fibrosis in systemic inflammatory disorders as well as useful clinical cases with implications for daily clinical practice.

Andrew Hassell from Keele University UK gives a - very well-considered, given his enormous experience - personal view on the Rheumatology training process in the UK, highlighting the positive and negative aspects of key developments in terms of selection, supervision, assessment and quality assurance of training that have taken place over the last 2 decades.^1^ Given the ongoing argument in Greece and other countries regarding changes and reorganization of training for Rheumatology trainees, this topical editorial can promote discussion and considerably contribute to the development of a fair and transparent system in favour of the national health system, rheumatology service delivery and physicians themselves.

Klavdianou et al.^2^ provide novel insights regarding the role of the canonical Wnt signaling pathway in the regulation of fibrotic process. It is known that Dickkopf-1 (Dkk-1) - an endogenous Wtn signaling pathway inhibitor - is involved in joint remodeling and represents a crucial player in the balance between joint destruction and new bone formation in rheumatoid arthritis and ankylosing spondylitis respectively, as well as in osteoporosis. Recent data indicate Wtn signaling as a mediator of transforming growth factor-beta (TGF-β) induced fibrosis suggesting that inhibition of this pathway via Dkk-1 may be an attractive therapeutic approach for halting fibrosis in several conditions.

Gurcay and Akinci^3^ review the utility of various types of physical therapy; such as electrical therapy, hydrotherapy, thermotherapy and therapeutic exercise in autoinflammatory diseases underlying for one more time the role of non-pharmacological therapeutic strategies in the overall management of systemic diseases and the necessity for an individualized treatment approach.

Apart from strictly clinical measurements, the modern management of lupus patients necessitates the assessment of parameters such as fatigue, sleep quality and overall health status. In an interesting article, Magro et al.^4^ describe the process of translating into Maltese language scoring algorithms for lupus individuals and how the questionnaires were validated by the patients. This has implications for many assessment tools if they are to be used in different languages.

The challenges of rheumatology care and research in modern Russia are presented by Elena Tchetina.^5^ She discusses the new trends in treatment strategies of rheumatic diseases, the development of a three-tier standardized system for the management of these conditions including amongst others treatment protocols, rehabilitation and patient education programs. Of note, T2T protocols for rheumatoid arthritis in Russia are based on the initiation of high doses subcutaneous methotrexate with considerably good outcomes. There are obvious opportunities for learning from each other’s experience in different countries and health systems, and for research and practice evaluation collaborations in the future, activities that MJR wants very much to support and facilitate.

Dr Clifopoulos discusses the effect of Vitamin-D levels on vascular morbidity and skin cancer risk concluding that there are no robust data supporting any direct and causal relationship between these conditions.^6^ This mini review concludes with some valuable points weighing up sun exposure as a source of Vitamin-D and risk factor for skin cancer from a dermatology perspective.

Prof Sakkas comments on emerging data from double-blind controlled studies published a couple of months ago suggesting that tofacitinib - an oral Janus kinase inhibitor- may represent an alternative treatment option for either biologic naïve or TNF-a inhibitor exposed patients with psoriatic arthritis who are unresponsive to methotrexate.^7^

In this issue, you can also find a couple of interesting cases discussing common clinical problems; such as diagnostic uncertainties in anti-synthetase syndrome,^8^ difficulties in treating complicated patients with biologic drugs and how to overcome these problems^9^ as well as clinical images from a lupus patient with significant cardiac involvement.^10^

Last but not least, we are very happy to host in this issue a nice historical note by Tsoucalas and Sgantzos who remind the very first – almost forgotten and underappreciated - description of rheumatoid arthritis back in early 19^th^ century proposed by French physician Landré-Beau-vais.^11^

Finally, we would like to express our sincere gratitude to the reviewers – listed in the current issue - who provided their judgment concerning the quality of the submitted papers and have enormously contributed to the progress of the *MJR* in 2017.
